# HIV Antiretroviral Medication Neuropenetrance and Neurocognitive Outcomes in HIV+ Adults: A Review of the Literature Examining the Central Nervous System Penetration Effectiveness Score

**DOI:** 10.3390/v14061151

**Published:** 2022-05-26

**Authors:** Alyssa Arentoft, Katie Troxell, Karen Alvarez, Maral Aghvinian, Monica Rivera Mindt, Mariana Cherner, Kathleen Van Dyk, Jill Razani, Michaela Roxas, Melissa Gavilanes

**Affiliations:** 1 Department of Psychology, California State University, Northridge, Los Angeles, CA 91330, USA; karen.alvarez.429@my.csun.edu (K.A.); jill.razani@csun.edu (J.R.); michaela.roxas.118@my.csun.edu (M.R.); 2 Department of Neuropsychology, Pacific Neuroscience Institute Foundation, Santa Monica, CA 90404, USA; katie.troxell2@gmail.com; 3 Department of Psychology, Fordham University, Bronx, NY 10458, USA; maghvinian@fordham.edu (M.A.); riveramindt@fordham.edu (M.R.M.); 4 Department of Neurology, Icahn School of Medicine at Mount Sinai, New York, NY 10029, USA; 5 Department of Psychiatry, University of California, San Diego, CA 92093, USA; mcherner@ucsd.edu; 6 Semel Institute for Neuroscience and Human Behavior, University of California, Los Angeles, CA 90095, USA; kvandyk@mednet.ucla.edu; 7 Department of Psychology, Pepperdine University, Malibu, CA 90263, USA; melissa.gavilanes@pepperdine.edu

**Keywords:** HIV, neuropenetrance, CNS, central nervous system penetration effectiveness, CPE, ARV medications, antiretrovirals, CART, neuropsychological functioning, neurocognitive outcomes

## Abstract

This literature review summarizes the existing research examining the CNS penetration effectiveness (CPE) score and neurocognitive outcomes (i.e., neuropsychological assessment and neurocognitive screening) in HIV+ individuals. Despite the effectiveness of Combined Antiretroviral Therapy (CART) in reducing mortality and morbidity in HIV and controlling viral replication, HIV often persists in the Central Nervous System (CNS), and rates of neurocognitive impairment remain higher than predicted in the post-CART era. The CPE score was developed to rank antiretroviral regimens on their ability to penetrate the CNS and potency in inhibiting the virus, and it has been examined in relation to neurocognitive functioning for over a decade. Based on the results of 23 studies, we conclude that CPE is not as strongly associated with neurocognitive outcomes as initially hypothesized, although higher CPE ARV regimens may be associated with modest, improved outcomes in global neurocognitive functioning, and to a lesser extent attention/working memory and learning/memory. Conclusions, however, are limited by the heterogeneity in study design and methods, and the lack of a more recent CPE metric update. It is recommended that future research in this area employ comprehensive, standardized neuropsychological test batteries and examine domain-level performance, and use the newer 2010 CPE metric, although an updated CPE ranking is urgently needed.

## 1. Introduction

Antiretroviral (ARV) medications are first-line treatments for HIV+ individuals and are key to managing HIV disease. ARV medications reduce viral replication that would otherwise allow the disease to rapidly proliferate throughout the body. They have greatly improved morbidity and mortality rates among individuals with HIV/AIDS, particularly since the inception of highly active antiretroviral therapy (HAART) in 1996, now referred to as Combined Antiretroviral Therapy (CART) [1,2]. CART combines multiple antiretroviral medications in one regimen and has been found to provide more potent, effective control of the HIV virus than earlier, single-medication regimens. Before the advent of antiretroviral medications, high viral loads and CD4 counts were associated with more frequent and severe disorders; CART has helped to reduce the incidence of these conditions and improve survival rates [3]. With CART, life expectancy among HIV+ individuals has increased by over 20 years, and is continuing to increase over time [4]. Overall, improvements in the pharmacological treatment for the human immunodeficiency virus (HIV) has drastically changed the face of the HIV epidemic over the last few decades, transforming a once fatal disease into a chronic illness.

Despite the effectiveness of CART in suppressing the HIV virus, medications cannot completely eradicate the virus from the system and there is still no cure for this disease. The HIV virus has an affinity for the central nervous system (CNS) [5], and studies have shown that even with stable CART treatment, latent viral reservoirs persist in the CNS, including the cerebrospinal fluid or CSF [2]. The presence of the virus in the brain can trigger a cascade of events implicated in neuropathogenesis. These processes particularly affect frontostriatal and subcortical circuitry, and the resulting neuropsychological and functional declines are characterized as HIV-Associated Neurocognitive Disorders (HAND) [6,7,8,9,10,11]. Although patterns of neuropsychological impairment are variable, deficits are often reported in processing speed, attention, executive functioning, learning, and memory [6,12,13,14,15,16,17].

Even in the post-CART era rates of cognitive impairment remain high, with an estimated 50% of HIV+ individuals exhibiting HAND [6] including those with long-standing viral suppression [17]. While rates of severe impairment associated with uncontrolled HIV proliferation (e.g., HIV-associated Dementia (HAD)) have decreased, mild and moderate forms of neuropsychological impairment have increased. Thus, neurocognitive decline remains a critical issue in this population [18,19,20,21]. It is unclear why cognitive impairments persist. Some studies have found that comorbid conditions such as vascular disease may more strongly predict HAND diagnosis than HIV RNA concentrations [22]. One question that surfaced is whether and how newer and more potent HIV antiretroviral medications affect the brain. Antiretroviral CNS neuropenetrance—also referred to as “CNS neuropenetrance”, which includes a medication’s ability to cross the blood–brain barrier (BBB) and penetrate the CNS—has been considered. Some HIV medications may be better at controlling the virus in the periphery than in the CNS, which is referred to as lower CNS efficacy. While many have suggested that ARV medications with higher neuropenetrance may protect the brain by more potently inhibiting the virus, some have questioned whether more neuropenetrant medications may be neurotoxic, thereby further damaging the brain. This has led to increased interest in the dynamics of CART pharmacology, including differences in blood–brain-barrier (BBB) penetration, and to the methods for classifying ARV neuroactivity or neuropenetrance.

Currently, the most widely used method for classifying antiretroviral neuropenetrance is the CNS penetration effectiveness (CPE) rank. Developed by Letendre and colleagues, this hierarchical scheme numerically ranks antiretroviral regimens on their ability to penetrate the CNS. Numerical rankings are derived from pharmacological data, particularly the pharmacokinetic, chemical, and pharmacodynamic properties of each ARV medication [23,24]. Lower CPE medications are thought to less potently inhibit the virus and allow larger viral reservoirs to remain in the brain, leading to more viral replication. Research has shown that higher CPE medications have been associated with lower viral load in both cerebrospinal fluid (CSF) and plasma [23,25,26]. CPE has also been associated with structural changes in the brain; for example, CPE 2010 score was specifically associated with reduced gray matter integrity among patients in the very early stages of HIV (i.e., less than 1 year duration) [27]. Although CPE score has been calculated and examined in HIV research for over a decade, there is currently no review of the literature examining the CPE metric and neurocognitive outcomes (i.e., neuropsychological assessment and cognitive screening) in HIV+ individuals. Therefore, the purpose of this review is to summarize the existing literature and provide conclusions about the evidence regarding CPE score and neurocognitive outcomes among adults living with HIV.

As described above, the CPE rank is a summary score developed by Letendre and colleagues that hierarchically ranks HIV medications on their CNS penetrance or neuropenetrance based on pharmacological data and physical properties. The CPE metric was first described in 2008 [23] in which medications were assigned a 0–2 score, with higher scores reflecting greater neuropenetrance. Specifically, scores of 0 were defined as no or low neuropenetration, scores of 0.5 suggest an intermediate-level of neuropenetration, and scores of 1.0 reflect adequate or good neuropenetration. This was later revised, and the 2010 CPE metric updated by Letendre and colleagues assigns each HIV medication a 1–4 integer score, with higher scores again reflecting greater neuropenetrance [24]. This update provides CPE ranks for a wider variety of HIV medications, including newer medications, and also allows for finer distinctions between degrees of neuropenetrance through its increased range. Per convention, scores are summed for all HIV medications in a patient’s regimen [25]. The CPE scores for commonly prescribed ARV medications, for both the 2008 and 2010 CPE metrics (see Supplementary Materials, Table S1). For a more detailed description of the development and update of the CPE rank, please see [23,24].

In comparing the two ranking systems, Ciccarelli and colleagues [28] found that the 2010 ranking system is more effective and associated with better neurocognitive performance. Similarly, CPE 2010 was significantly associated with ventricular atrophy in HIV+ individuals when CPE 2008 was not [29]. More recent studies typically use the updated 2010 metric; however, CPE 2008 has historically been investigated more frequently. Studies using either CPE 2008 or CPE 2010 were included in this review. For each article reviewed, the CPE ranking used, study and sample characteristics, and key methodology are presented in our summary table (see Supplementary Materials, Table S2). Results and conclusions are described in the text below.

## 2. Materials and Methods

### Search Strategy and Selection Criteria

We first identified studies that cited either 2008 or 2010 CNS Penetration Effectiveness score [23,24], and these were examined for inclusion. Second, literature searches were conducted using the following search terms in PubMed, Google Scholar, and PsycINFO: “CNS Penetration Effectiveness”, “CNS Neuropenetrance”, “CPE score”, “ARV neuropenetrance”, “antiretroviral neuropenetrance”, “ARV efficacy” and “antiretroviral efficacy”. These search terms yielded a total of 3899 articles. From these results, studies that did not examine HIV+ adults, antiretroviral neuropenetrance measures (i.e., CPE 2008 or CPE 2010 score), and neurocognitive outcomes were excluded. Only studies that specifically examined the relationship between CPE 2008 or CPE 2010 scores and neurocognitive performance (i.e., neuropsychological tests or cognitive screenings) were included. These articles were independently reviewed and summarized by at least two study authors (MA, KA, MG, and/or AA). Study quality was determined by the first author using the Joanna Briggs Institute (JBI) Critical Appraisal Checklist for Systematic Reviews and Research Syntheses (JBI, 2020). Ultimately, this yielded a total of 23 studies which were included in this review.

## 3. Results and Discussion

The following review summarizes the existing literature on the neuropenetrance of HIV medication regimens as assessed by CPE score and observed neurocognitive outcomes in HIV. First, we provide background on how CPE affects plasma and CSF viral load and the literature that has examined these relationships in patients that are initiating HIV medication and patients on existing ARV regimens. Next, we focus specifically on the literature examining the relationship between CPE and neuropsychological test performance or neurocognitive screening, considering global neurocognitive outcomes first and then discussing the available results by traditional neuropsychological domain. Finally, we provide our overall conclusions about the CPE literature in this area and some general recommendations for future research and practice.

### 3.1. CPE and Viral Load in Plasma and CSF

The main goal of antiretroviral therapy in patients with HIV, particularly combined antiretroviral therapy (CART), is to suppress viral replication and reduce the amount of the virus in both plasma and cerebrospinal fluid (CSF). By suppressing viral replication, it is thought that CART reduces the amount of HIV in the brain, which in turn reduces damage to the brain and central nervous system that would result in neurocognitive dysfunction [30]. Therefore, it is expected that medications with higher CPE rank would more effectively reduce the amount of the virus in plasma and CSF. CSF measurements are used as proxies for ARV concentrations in the brain, since more direct measurement in tissue can only be performed post-mortem; however, it is important to note that CSF and brain concentrations may differ, and in fact, some studies suggest that CSF measurements may underestimate concentrations in the brain [31,32,33]. This is a limitation of both CSF measurement as well as the CPE metric.

Prior to the development of the CPE score, researchers suggested that the neuropenetrance of HIV medications may be important. Sacktor and colleagues [30] studied plasma and CSF viral loads at baseline and follow-up in groups with single and multiple CNS-penetrating ART medications, and found that adding at least one antiretroviral mediation with good CNS penetration can aid in suppressing the virus both in CSF and plasma [30]. When this was initially examined using the CPE ranking system, some studies did not find these effects in ARV-naive patients. Tozzi and colleagues [34] found no association between CD4 cell counts, plasma viral loads, and CPE scores. Similarly, Marra and colleagues [35] found no significant association between CPE rank, number of ARV medications, and viral suppression. In a study specifically comparing ARV-naïve and ARV-experienced patients, they found that ARV-naïve patients had greater odds of plasma and CSF viral suppression compared to ARV-experienced patients. In terms of CPE rank and suppression, there were greater odds of CSF HIV RNA suppression when the 2008 CPE score was greater than or equal to two; however, there was no association between plasma viral suppression and CPE rank.

However, later research has shown that ARV regimens that are modified in ARV-experienced HIV patients may provide benefits. A study specifically examining 2008 CPE ranks and viral load found that CSF HIV RNA was significantly lower in those on stable ARVs compared to those not on ARVs [36]. Additionally, they found that higher CPE scores were significantly correlated with improved CSF viral suppression. Similarly, Cusini and colleagues [37] found that higher CPE scores were associated with better HIV RNA viral suppression using both 2008 and 2010 CPE ranks. They reported that a minimum threshold score of 2 under the 2008 CPE rankings and a minimum threshold score of 7 under the 2010 CPE rankings achieved the most successful viral suppression [37].

For some patients with HIV, HIV RNA is detectable in the CSF even though plasma viral suppression has been achieved, which is referred to as “CSF viral escape” [38]. It is thought that between 4–20% of ARV-experienced HIV patients experience CSF viral escape [38]. Even when plasma viremia is suppressed by ARVs, HIV RNA may still replicate if poor CSF penetration causes low concentrations of the medications in the CSF, which can lead to drug resistance. It has been hypothesized that this could result in neurological symptoms, even when plasma viral load is suppressed. Results suggest that regimens with lower CPE scores may increase the likelihood of viral escape. Canestri and colleagues [39] examined ARV-experienced patients with HIV-related neurological symptoms who had discordant plasma and CSF RNA. ARV regimens were modified based on drug resistance and that no patient had a CPE rank of less than two. Within six weeks, they found neurological improvement, normalization of CSF cellularity and protein levels, and undetectable HIV viral loads [39]. Over the course of a four-year follow-up, approximately 7% of patients experienced viral escape [38]. When CPE scores were adjusted for resistance mutations, adjusted CPE scores were lower in those experiencing viral escape, although raw CPE scores between the viral escape and non-escape group were not significantly different. Furthermore, differences between plasma and CSF viral load concentrations suggest that HIV compartmentalization can occur [40]. Viral sequences may become genetically distinct across tissue compartments in the CNS and periphery, and differences in the concentration and movement of ARV medications in the CNS may be important to address. ARV medications may insufficiently target particular sequences and/or compartments, and low CPE medications may be more likely to do so, but this should be investigated further.

Moreover, although antiretroviral inhibitory concentrations have been found to correlate with CPE scores, variability in CSF viral load across individuals has still been observed, suggesting that some patients may receive a greater benefit from ARV medications than others [41,42].

### 3.2. CPE and Neurocognitive Functioning

While previous studies have broadly reported associations between higher CPE scores and improved CSF and plasma viral suppression, studies examining the association between CPE and neurocognitive outcomes have had more variable results. Below, we summarize findings from studies that explored the relationship between CPE rank and neurocognitive functioning, organized by neurocognitive domain. Of note, no studies specifically examined visuospatial functioning (although some included visuospatial measures in their global neurocognitive summary scores), so this domain is not discussed.

#### 3.2.1. Global Functioning

Global neurocognitive functioning typically provides a summary of neurocognitive abilities by averaging data obtained across several neurocognitive domains; this can vary from a more comprehensive summary, obtained from several tests across all major neuropsychological domains (e.g., learning/memory, attention, processing speed, executive functioning, motor functioning, and verbal functioning), to a less comprehensive summary, examining one or a few tests across a few traditional domains. In the extant literature examining CPE and neurocognitive performance, most studies have focused on global functioning and have used abbreviated screening batteries, which is a limitation. Additionally, some studies examine global neurocognitive performance as a continuous measure while some use these measures to provide HAND diagnoses, typically employing the Frasati criteria [6]. However, as a statistically derived approach, some have criticized the Frascati criteria, suggesting that the recommended one standard deviation cut-off for impairment may lead to a small percentage of false positives in HAND diagnoses [43], although others have empirically supported it for its validity and sensitivity [44]. Of the 15 studies examining global functioning, generally, the weight of the evidence seems to suggest a modest relationship between CPE and global neurocognitive performance.

First, the evidence from cross-sectional research is somewhat more consistent. In a large sample of relatively healthy and cognitively intact HIV+ individuals (i.e., about 75% of the sample performed within one standard deviation of the mean) on stable ARV treatment for at least 90 days, higher CPE 2010 score was significantly associated with lower global deficit score (i.e., better global neurocognitive functioning) based on a screening of neuropsychological tests. The likelihood of neurocognitive impairment decreased by 17% for each point increase in CPE 2010 score [45]. In a study comparing HIV-RNA suppressed patients with ARV-naive patients commencing their initial regimens, 2010 CPE scores were associated with neurocognitive impairment in patients with nadir CD4 below 200; specifically, immunocompromised patients with lower CPE scores were more likely to be diagnosed with a HAND than those with higher CPE scores. Global functioning in this study, however, was based on a 4-test screening battery of motor and processing speed tests. Among all patients, those with CPE scores less than seven had worse neuropsychological functioning, although this only reached trend-level [46]. Using a more comprehensive neuropsychological battery, including all traditional neuropsychological domains, 2010 CPE scores were independently associated with better neurocognitive performance, and patients with higher 2010 CPE scores were less likely to be diagnosed with HAND, although this relationship was not observed for 2008 CPE scores [28]. Fabbiani and colleagues [47] conducted a retrospective examination of HIV+ individuals who had received several neuropsychological tests during outpatient care, and obtained data on viral resistance, which would impact the effectiveness of the ARV regimen. Initially, they found no relationship between CPE 2010 scores and global neuropsychological functioning, but after correcting for viral resistance, they found that higher CPE scores were associated with significantly lower risk of global neuropsychological impairment [47]. Finally, higher CPE 2008 score was significantly associated with better global neuropsychological functioning based on a comprehensive battery at 40-month follow-up, but not at 20-month follow-up, in a sample of HIV+ individuals with cognitive symptoms or CD4 count below 200 [34]. Furthermore, these associations were found at both 20- and 40-month visits among patients who were cognitively impaired and among those who were virally suppressed. Simioni and colleagues [17] examined HAND diagnoses based on the results of a neurocognitive screening in HIV+ individuals with suppressed viral load who reported cognitive complaints. In this study, CPE 2008 score did not significantly differ between those who received any level of HAND diagnosis and those who did not. However, a trend was observed such that patients with CPE scores of at least 2 were less likely to be given a HAND diagnosis involving functional impairment compared to patients with lower CPE scores [17]. Wright and colleagues [48] found no association between average global neuropsychological performance and 2008 CPE scores among HIV+ individuals with CD4 counts higher than 350, although this was based on z-scores obtained from a 5-test screening battery focusing on motor functioning and processing speed. Similarly, based on a 4-test screening battery, global NP functioning was not associated with 2010 CPE scores when examined categorically (i.e., CPE < 7 vs. CPE ≥ 7) or when examined continuously [49]. Finally, among older HIV+ individuals on stable CART for at least 6 months, CPE 2010 was not significantly associated with global neurocognitive performance, although a different estimate of ARV effectiveness in inhibiting monocytes (i.e., monocyte efficacy) was significantly associated [50]. Monocyte efficacy is linked to, and significantly associated with CPE, but has not been investigated in other studies. Participant age, however, may be important; the ability to absorb, distribute, metabolize, and excrete medications may become less effective, which may affect the relationship between CPE and neurocognitive functioning. Additionally, older HIV+ patients tend to have more comorbidities, and may be taking a larger number of medications overall, which increases the likelihood of side effects and other interactions [51].

Several longitudinal studies have also found higher CPE to be associated with improved global neurocognitive functioning. Among HIV+ individuals assigned to a CART regimen (i.e., ARV-naive individuals or those experiencing treatment failure), CPE 2008 scores ≥ 2 predicted improvement in global cognitive functioning over 48 weeks, with the most marked improvement seen at 12 weeks. In multivariate analyses, only higher CPE and worse baseline NP were significant predictors of NP improvement, while longitudinal decreases in viral load were not [52]. Similarly, Vassallo et al. [53] found that patients with lower CPE 2010 scores at baseline were significantly more likely to be diagnosed with HAND based on neuropsychological test performance. At 2-year follow-up, patients with lower CPE scores were more likely to have cognitively deteriorated, meaning that they were diagnosed with HAND when they had not been at baseline, or had progressed to a more severe HAND diagnosis. In a sample from a large clinical trial of HIV+ individuals with suppressed HIV viral load, better global neuropsychological scores were associated with higher 2008 CPE rankings in participants taking more than three ARV medications, but not in patients taking less than three ARVs, even after accounting for numerous potential covariates [54]. A small sample of HIV+ individuals experiencing at least mild cognitive symptoms who were on CART with undetectable viral load for at least one year received ARV intensification, meaning that ARV medications were changed or added to their regimen in order to increase the overall regimen CPE score [55]. CPE scores were adjusted for drug resistance and increased an average of 4 points across the sample. Participants received a comprehensive neuropsychological battery used to generate HAND diagnoses and global deficit scores (GDS), and after 48 and 96 weeks, higher CPE score was associated with greater improvement in neurocognitive functioning. Participants, however, were not randomized and there was no comparison group (i.e., participants not receiving ARV intensification). Patients also had very lengthy past treatment histories and exhibited significant medication resistance.

Other longitudinal studies have failed to replicate this relationship, although several also had some noteworthy limitations. In a randomized clinical trial, HIV+ participants who were assigned to the higher CPE medication arm (based on CPE 2008 score) showed a greater reduction in global cognitive deficits over the course of 16 weeks, based on a comprehensive neuropsychological battery, although the difference between groups was not statistically significant [56]. This effect was most pronounced among individuals with plasma HIV viral load suppression and those who were previously ARV-naive, but again, not statistically significant. However, the authors note that conclusions were limited by the fact that the RCT was discontinued early due to limited recruitment, cognitive changes were assessed over just 16 weeks, and treatment groups differed on several key characteristics that may affect the results (e.g., nadir CD4, hepatitis co-infection, plasma HIV viral load). An evaluation of the large-scale multisite CHARTER study baseline cohort showed that CPE 2008 score did not emerge as a significant, independent predictor of global deficit score, derived from a comprehensive neuropsychological test battery, when included in a multivariate logistic regression [20]. In a subset of the CHARTER longitudinal sample, HIV+ individuals were classified based on whether they improved, declined, or remained stable based on global neurocognitive performance over an average of 35 months, obtained from a comprehensive NP battery applying regression-based norms. There were no significant differences observed between these groups on CPE 2008 score [57]. Marra and colleagues [35] found no significant relationship between impaired global cognitive scores on a short screening of neurocognition and CPE 2008 score at baseline. Unexpectedly, participants with a CPE score of ≥2 had lower global neurocognitive scores over the course of the one-year study. It was suggested that worse cognitive performance in participants with better neuropenetrance could reflect the neurotoxic effects of high CPE medications in participants with advanced HIV disease [35], a hypothesis that has not yet seen further empirical support. Additionally, Cross and colleagues [58] did not find differences in global cognitive performance, based on a comprehensive neuropsychological test battery, between patients with high and low 2010 CPE score ARV regimens. Interestingly, however, South Africa (where this study was conducted) changed their recommended first-line ARV medications from a higher to a lower CPE regimen, which may be an important difference between this and other samples.

Although findings have differed across studies, generally, there seems to be a modest relationship between higher CPE scores and better global neurocognitive performance. Studies that employed the newer, 2010 CPE ranking system and those employing comprehensive neuropsychological batteries were more likely to report significant findings, suggesting that methodology may be at least one important factor. Differing patient characteristics, including length of time on ARV therapy, virological characteristics and immunological status, are all further complicating factors.

#### 3.2.2. Attention/Concentration and Working Memory

In contrast to global functioning, fewer studies have specifically examined domain level neuropsychological or neurocognitive performance. The five studies that have examined attention, working memory, and/or concentration and CPE score are somewhat equivocal, but suggest that there may be a modest relationship between these variables.

A higher CPE 2008 score was significantly associated with better concentration at both 20- and 40-month follow-up, in a sample of HIV+ individuals with cognitive symptoms or CD4 count below 200 [34]. This relationship was also significant among those who were cognitively impaired (at 20- and 40-month visit) and those who were virally suppressed (at 20-month visit). In a large cohort of cognitively intact HIV+ individuals on stable ARV treatment for at least 90 days, CPE 2010 score was significantly associated with better working memory (i.e., WAIS-R Digit Symbol; [45]. Ciccarelli and colleagues reported that better performance on one measure of attention/working memory (i.e., double barrage test) was associated with higher 2010 CPE score [28], although this is a less commonly used neurocognitive measure. However, in a retrospective study, CPE 2010 scores were not associated with attention (i.e., digit span subtest), even after correcting for viral resistance, in HIV+ patients receiving routine outpatient care [47]. Among a small cohort of HIV+ individuals with undetectable viral load on stable CART, CPE 2008 score did not correlate with attention and working memory (i.e., Digit Symbol Substitution Task (DSST) and WAIS-IV Letter-Number Sequencing) at baseline or at 2-year follow-up [59]. However, this virologically well-controlled sample also did not show significant neurocognitive decline over time, and neuropsychological performance was not associated with other virological characteristics. The authors note that CPE range was also limited, with most participants receiving relatively neuropenetrant medications.

#### 3.2.3. Processing Speed

Out of six studies that have specifically examined the domain of processing speed, the evidence is more consistent. There does not appear to be a significant relationship between processing speed and CPE score.

Among HIV+ individuals exhibiting cognitive symptoms or immunosuppression (e.g., CD4 count below 200), higher CPE 2008 scores were significantly associated with better processing speed at 20- and 40-month follow-up visits [34]. This relationship was also significant among those who were cognitively impaired (at 20- and 40-month visit) and those who were virally suppressed (at 20-month visit). However, this is the only study in our review that reported such a relationship. No significant differences in CPE 2008 scores were found between HIV+ patients classified as cognitively impaired (i.e., performance below the 10th percentile on at least three tests compared to HIV-negative control group) and those who were not impaired on the WAIS-III Digit Symbol Coding subtest [60]. The potential limitations of the definition of impairment were described above. A retrospective study showed that CPE 2010 scores were not associated with processing speed (i.e., WAIS Digit Symbol), even after correcting for viral resistance, in HIV+ patients receiving routine outpatient care [47]. Similarly, CPE 2010 score was not significantly associated with processing speed (i.e., WAIS Digit Symbol) in a large cohort of cognitively intact HIV+ individuals on stable ARV treatment for at least 90 days [45]. In a cross-sectional study employing a screening of neuropsychological functioning, processing speed (i.e., Trailmaking Test-Part A or TMT-A) was not associated with 2010 CPE scores when examined categorically (i.e., CPE < 7 vs. CPE ≥ 7) or when examined continuously [49]. Finally, among HIV+ individuals with undetectable viral load on stable CART, CPE 2008 score did not correlate with processing speed (i.e., Trailmaking Test-Part A or TMT-A) at baseline or at 2-year follow-up [59]. As mentioned above, neuropsychological performance was also not associated with other virological characteristics, and participants tended to be prescribed high CPE regimens.

#### 3.2.4. Learning/Memory

The relationship between CPE score and learning and/or memory, measured through either verbal and/or visual tests, has been examined in 10 studies. Although some studies did find a significant relationship between learning/memory and CPE score, generally, the relationship is not consistently reported. Studies that did, however, employed the newer, 2010 CPE score.

In a large group of cognitively intact HIV+ individuals on stable ARV treatment for at least 90 days, CPE 2010 score was significantly associated with better learning/memory (i.e., HVLT-R and BVMT-R) [45]. Ciccarelli and colleagues [28] similarly found that participants with higher 2010 CPE scores were significantly less likely to be impaired on verbal memory (i.e., Rey Auditory Verbal Learning Test, or RAVLT), and notably, no significant association was found for 2008 CPE scores. No relationships were reported for measures of visual memory (i.e., Rey Complex Figure Test or RCFT) in this study; however, in another study, Keutmann and colleagues found that higher 2010 CPE scores were associated with better visual memory (i.e., BVMT-R) performance [61]. Of note, this measure employed by Keutmann and colleagues is less confounded by executive functioning than the RCFT, which was used by Ciccarelli. In another study examining RAVLT, CPE 2010 scores were not associated with verbal memory (i.e., RAVLT) in HIV+ patients receiving routine outpatient care, although a trend was observed after correcting for viral resistance [47]. No significant differences in CPE 2008 scores were reported between HIV+ patients who were cognitively impaired and those who were not on a test of verbal memory (i.e., Botswana Auditory Verbal Learning Test, BAVLT; Lawler et al., 2011). The cut-off used to determine neurocognitive impairment, however, may have been overly conservative; the threshold was defined as performance below the 10th percentile compared to an HIV-negative control group. In a cross-sectional study employing a screening of neuropsychological functioning, verbal learning (i.e., HVLT-R, which is the verbal analog to the visual BVMT-R) was not associated with 2010 CPE scores when examined categorically (i.e., CPE < 7 vs. CPE ≥ 7) or when examined continuously [49]. CPE 2008 score was not significantly associated with better memory performance at either 20- and 40-month visit in a sample of HIV+ individuals with cognitive symptoms or CD4 count below 200 [34], although the specific memory measure used was not reported. In a small sample, CPE 2008 score was not significantly associated with performance on a brief, less commonly used screening of memory (i.e., the “5 words” test) among HIV+ individuals on a stable CART regimen for 4–7 years [62]. Among HIV+ individuals with undetectable viral load on stable CART, CPE 2008 score did not correlate with verbal learning and memory (i.e., HVLT-R) at baseline or at 2-year follow-up [59]. The limitations of this study were mentioned above, but briefly, neuropsychological performance was also not associated with other virological characteristics, and participants tended to be prescribed high CPE regimens. Finally, no significant differences were reported between participants with high vs. low 2010 CPE scores based on a median split, although two less commonly used measures of procedural learning were employed in this study (i.e., weather prediction and pursuit motor) [63].

#### 3.2.5. Executive Functioning

Executive functioning has been examined in five studies. Similar to the findings reported in several other domains, studies have generally have not reported a significant relationship between CPE and executive functioning. Studies tended to include only a single, brief screening of executive functioning, which may be insufficient given the heterogeneity and complexity of this neuropsychological domain. Specifically, most studies used a trailmaking-type test, which also overlaps significantly with other NP domains.

A higher CPE 2008 score was significantly associated with better mental flexibility at both 20- and 40-month follow-up, in a sample of HIV+ individuals with cognitive symptoms or CD4 count below 200 [34]. In a cross-sectional study employing a screening of neuropsychological functioning, performance on a measure of executive functioning (i.e., TMT-B) was not associated with 2010 CPE scores when examined categorically (i.e., CPE < 7 vs. CPE ≥ 7) or when examined continuously [49]. Similarly, among HIV+ individuals with undetectable viral load on stable CART, CPE 2008 score did not correlate with executive functioning (i.e., TMT-B) at baseline or at 2-year follow-up [59]. As mentioned previously, participants were generally on high CPE regimens and NP performance was not associated with other virological characteristics. One study concluded that CPE 2008 scores were not associated with performance on an executive function task (i.e., Color Trails 2; [60]. Finally, in a small sample, higher CPE 2008 score was associated with worse performance on a short screening of executive functioning (i.e., Frontal Assessment battery) among HIV+ individuals on a stable CART regimen for 4–7 years [62].

#### 3.2.6. Motor Functioning

Motor functioning has been examined individually in only 3 studies, making this the least investigated domain in our review. It was not found to be significantly associated with CPE. Notably, however, most studies that focused on global neurocognitive functioning included measures of motor functioning and tended to use the same, widely used tests: either grooved pegboard [20,35,46,48,52,58] and/or finger tapping test [35,48,53,58]. Unfortunately, however, these studies did not examine CPE and motor functioning at the domain level.

CPE 2010 scores were not significantly associated with motor functioning (i.e., grooved pegboard) in HIV+ patients receiving routine outpatient care, although a trend was observed after correcting for viral resistance [47]. No significant difference in CPE 2008 scores were found between HIV+ patients classified as cognitively impaired (i.e., performance below the 10th percentile compared to HIV-negative control group) and those who were not on grooved pegboard [60]. Finally, CPE 2008 score was not significantly associated with motor functioning at either 20- and 40-month visits, although higher CPE 2008 score was significantly associated with better motor functioning among those who were cognitive impaired (at 20- and 40-month visit) and those who were virally suppressed (at 20-month visit) [34].

#### 3.2.7. Language/Verbal Functioning

Language functioning has been examined in four studies, none of which reported a significant association with CPE score. Of note, all studies used screening of verbal fluency to assess language/verbal functioning, and similar measures were employed across studies. While these verbal fluency measures are often combined with other tests in comprehensive batteries to examine attention/executive function, here, we will consider them in isolation as measures of language/verbal functioning.

Verbal fluency (i.e., COWAT semantic fluency) was not associated with 2010 CPE scores when examined categorically (i.e., CPE < 7 vs. CPE ≥ 7) or continuously [49]. CPE 2010 scores were not significantly associated with verbal fluency (i.e., COWAT letter fluency) in HIV+ patients receiving routine outpatient care, even after correcting for viral resistance [47]. No significant difference in CPE 2008 scores were found between HIV+ patients classified as cognitively impaired (i.e., performance below the 10th percentile compared to HIV-negative control group) and those who were not on a measure of verbal fluency (i.e., action fluency) [60]. Finally, among HIV+ individuals with undetectable viral load on stable CART, CPE 2008 score did not correlate with verbal fluency (i.e., letter fluency and action fluency) at baseline or at 2-year follow-up [59]. Overall, there is no evidence to date of a relationship between language or verbal functioning and CPE.

### 3.3. Summary of Confounding Factors to Consider

There are numerous factors that may potentially limit the conclusions that can be drawn from CPE and neurocognitive test performance based on their relationship with these variables or the degree to which they vary across studies. To summarize, the following confounds should be considered in examining and planning research in this area:

- History of ARV experience (e.g., ARV naïve prior to study enrollment or not; duration of ARV treatment; number of past ARV regimens, neuropenetrance of past ARV regimens; adherence)
- ARV drug resistance, inter-individual and intra-individual variability in CNS ARV concentrations
- ARV neurotoxicity, pharmacokinetics
- HIV compartmentalization and distribution of medications in the CNS
- Study sample size and observed power
- CPE score selected (e.g., 2008 vs. 2010) and cut-offs used
- Cross-sectional vs. longitudinal design (including follow-up duration)
- Years conducted (e.g., cohort effects, changes in medication prescribing practives)
- Influence of demographic factors (e.g., age, gender, race/ethnicity) and existing comorbidities (e.g., vascular disease, hepatitis C, metabolic disorders)
- Influence of health and healthcare disparities
- Neurcognitive domains, measures, and normative data selected; methods of computing impairment and HAND diagnoses

## 4. Conclusions

CPE seems to be a valuable and meaningful metric for evaluating the quality of CART neuropenetrance, and has been significantly linked to important pharmacological outcomes in HIV+ individuals. CNS-penetrating antiretroviral regimens may reduce morbidity for HIV+ individuals compared to regimens with low penetration. CSF viral replication is most effectively controlled by ARV regimens with high CNS penetration, and treatment regimens with a high CPE score are important to achieve CSF HIV-1 RNA suppression. Early and consistent CART treatment can preserve or improve patients’ cognitive functioning, and higher CPE regimens seem to be slightly more likely to provide these benefits compared to lower CPE regimens.

There is some evidence that treatment regimens with higher CPE scores may be associated with modest, improved neurocognitive outcomes. Higher CPE score has been associated with preserved or improved global neuropsychological functioning in a number of studies, though not all. Several studies have also reported increased HAND diagnoses among patients with lower CPE regimens. Global neurocognitive functioning has been more thoroughly examined to date, and thus, these findings are given greater consideration. Nonetheless, CPE is not as strongly or consistently associated with NP performance outcomes as many in the field initially hypothesized. There is mixed evidence for a relationship with attention/working memory and learning/memory, but research has not consistently supported a relationship between CPE and other neurocognitive domains when examined specifically at the domain or individual test level. Studies that did find significant associations tended to use CPE 2010 score and/or employ more comprehensive and standardized neuropsychological tests, which we discuss further as a direction for future research. This is generally consistent with the conclusions drawn by Lin and colleagues [64]; in a review of clinical treatment options and neuroHIV-targeted ARV treatment, the relationship between neurocognitive outcomes and CPE score was briefly summarized and found to be inconsistent based on primarily observational studies.

The heterogeneity in the methods employed across studies complicates interpretations in the literature. Studies include different CPE rankings (2008 vs. 2010) and may employ them continuously or use categorical cut-offs that often differ across studies. Further complicating the picture, there are also changes that have occurred over time in antiretroviral prescription practices. Increasingly over the last two decades, CART is more likely to be started early on in the disease course when CD4 counts still remain high, integrase inhibitors are being used more commonly as first-line antiretroviral treatments, and, the most recent rising interest in “single tablet regimens” (STR) and long-acting injectables [65,66,67]. These changes systematically affect CPE research over time; for instance, while CPE 2010 is more likely to be associated with neurocognitive outcomes, the newer CPE metric also coincides temporally with important changes in ARV prescribing practices.

Studies also use a wide variety of different neuropsychological tests and normative data, and may examine those data continuously or categorically. Few studies employed comprehensive neuropsychological test batteries applying the best available normative data, and even fewer have examined individual neuropsychological domains specifically. The variety of normative data is further complicated by the variety of different countries in which CPE research has been conducted.

Importantly, research has not adequately investigated race/ethnicity in the context of CPE. No study to date has explored whether or not CPE scores differ across racial/ethnic groups and factors that may drive any potential discrepancies. This is especially pertinent in the USA, where there are well-documented health disparities and systemic disadvantages among minoritized racial/ethnic groups in HIV [68,69]. Particularly, individuals who identify as Black/African American and/or Hispanic/Latinx, minoritized groups are not only disproportionately affected by HIV disease, but also receive poorer quality HIV-related healthcare, including delayed treatment [70,71] and less access to HIV medications, particularly newer medications [72,73]. They also experience significantly higher morbidity and mortality [74], including higher rates of neuropsychological impairment. Yet this has not been investigated in the CPE literature, and in fact, many studies have not adequately described the racial/ethnic background of their research study participants. Future studies should ensure that the demographic characteristics of their sample are fully described, including race/ethnicity, and should also investigate whether or not CPE scores differ across racial/ethnic groups. This systematic bias may underlie some of the inconsistent findings in the literature.

It is also difficult to disentangle the effects of CPE from other factors. The literature is confounded by the fact that the populations investigated often have lengthy histories of past ARV medication use prior to the current regimen. Extensive, suboptimal antiretroviral treatment (i.e., medications from the pre-CART era) may be particularly problematic. Therefore, it is currently difficult to disentangle the effects of pre-CART medications from the question of neuropenetrance. This remains a critical limitation of the current literature. Moreover, when and why patients are placed on higher or lower CPE medications may systematically differ, and these populations may be disproportionately sampled across studies. Studies that focus on HIV+ individuals who have been treated exclusively with combined antiretroviral therapy regimens, especially individuals who have never experienced treatment failure, are needed. For instance, randomized clinical trials assigning patients medication based on CPE regimen will significantly differ from samples investigating patients with worse functioning or adherence who have been switched to a different medication regimen, which may differ from community samples of newly diagnosed HIV+ individuals. Therefore, the varied reasons why higher CPE medications are prescribed will likely complicate all research in this area, especially cross-sectional research, although this may be attenuated by clinical trials. In fact, others have highlighted the need for randomized clinical trials in this area [64]. Additionally, the impact of comorbid neurological conditions, other medical conditions, and immunological factors (e.g., cardiovascular disease, hepatitis co-infection, CD4 counts) is difficult to quantify. In general, such conditions may also contribute to worse neurological outcomes and vary across studies.

Treatment is, necessarily, individualized for HIV+ individuals, particularly in those with advanced HIV disease. This is a complicating factor when comparing studies, but there are some general conclusions that researchers have suggested for clinical practice. It is important for HIV+ individuals to initiate CART early to increase the odds of preserving long-term brain health. Providers and patients should also be aware that HIV+ individuals may continue to develop neurocognitive impairment despite treatment with CART. Although medication decisions are necessarily the purview of physicians in terms of neurocognitive outcomes alone, research seems to suggest that higher CPE regimens may better preserve neurocognitive functioning than lower CPE regimens, or may not have a significant effect. Similarly, others have reported that the literature generally supports the finding that higher CPE scores are associated with better CSF HIV viral suppression [64].

Based on this review, we recommend that studies employ the 2010 CPE ranking score, which differentiates medications more finely across a wider range of CPE scores and provides rankings for a wider variety of medications. This, however, is a temporary solution; since the last CPE update, more patients are being prescribed newer CART regimens with medications that have not yet been assigned CPE scores, such as Dolutegravir, Bictegravir, and Doravirine. As more of these patients are enrolled in research studies, it will preclude examining the relationship between these ARV medications and neuropenetrance in the future. Therefore, a CPE ranking update is urgently needed, particularly as the CPE 2010 metric is now over a decade old.

We strongly recommend that studies examining neuropsychological/neurocognitive functioning administer comprehensive neuropsychological batteries, in consultation with a Clinical Neuropsychologist, whenever possible, especially batteries that have been well-validated in HIV+ individuals, such as those used in the CNS HIV Antiretroviral Therapy Effects Research (CHARTER) and Multicenter AIDS Cohort Study (MACS) studies [20,75], and apply the best available normative data [76,77]. In clinical settings, comprehensive neuropsychological evaluations may not always be feasible, and in such cases, groups such as the Mind Exchange Working Group [78] have discussed the strengths and limitations of the available screening tools. Neurocognitive screening, at minimum, is now recommended for all HIV+ patients, regardless of reported symptoms [43,78]. It is important to note, however, that screening tools often lack the sensitivity to identify less severe forms of HAND and also lack the specificity of comprehensive, multi-domain batteries. Comprehensive neuropsychological evaluation remains the criterion standard in the diagnosis of HAND.

Furthermore, it is useful if researchers examine neuropsychological functioning not only at the global level but at the domain level as well; a more fine-grained analysis will help inform whether or not some domains are preferentially impacted by less potent viral inhibition, as there is already long-standing evidence that HIV-related neurodegeneration compartmentalization may preferentially affect frontostriatal regions of the brain. We also recommend that researchers consider continuous measures of CPE and NP, as categorical cut-offs often differ between studies (i.e., median split) and may be somewhat arbitrary in the larger context. Finally, they should be sure to describe the virological and demographic characteristics of their sample, including race/ethnicity, and investigate the association between CPE scores and these variables. Overall, these recommendations may improve the generalizability of future research in this area and may contribute to our understanding of HIV medication neuropenetrance and neurocognitive outcomes in HIV+ individuals.

## Data Availability

Not applicable.

## Supplementary Materials

The following supporting information can be downloaded at: https://www.mdpi.com/article/10.3390/v14061151/s1, Table S1: CPE 2008 and CPE 2010 scores for HIV Antiretroviral Medications; Table S2: Summary of articles examining CPE and neurocognitive performance. See attached document.
