# Supplementary material for: HIV Antiretroviral Medication Neuropenetrance and Neurocognitive Outcomes in HIV+ Adults: A Review of the Literature Examining the Central Nervous System Penetration Effectiveness Score
Source: Viruses. 2022 May 26;14(6):1151. doi: 10.3390/v14061151 (PMC9227894; doi:10.3390/v14061151)
Supplement: Supplementary file 1 [file viruses-14-01151-s001.zip › viruses-1698975-supplementary.pdf]

Table S1. CPE 2008 and CPE 2010 scores for HIV Antiretroviral Medications.

Medication	CPE 2008 Score	CPE 2010 Score
Abacavir	1	3
Amprenavir	0.5	N/A
Amprenavir-r	1	N/A
Atazanavir/r	0.5	2
Darunavir-r	0.5	3
Delavirdine	1	3
Didanosine	0	2
Efavirenz	0.5	3
Emtricitabine	0.5	3
Enfuvirtide	0	1
Etravirine	N/A	2
Fosamprenavir	0.5	2
Fosamprenavir-r	1	3
Indinavir	0.5	3
Indinavir-r	1	4
Lamivudine	0.5	2
Lopinavir-r	1	3
Maraviroc	1	3
Nelfinavir	0	1
Nevirapine	1	4
Ritonavir	0	1
Raltegravir	0.5	3
Saquinavir/r	0	1
Stavudine	0.5	2
Tenofovir	0	1
Tipranavir-r	0	1
Zalcitabine	0	1
Zidovudine	1	4

Table S2. Summary of articles examining CPE and neurocognitive performance. See attached document.

Article	*N*	Study Type	Location	Years	Participant Demographics	HIV Duration	HIV characteristics	ARV history	CPE score used	CPE analysis	NP Domains and Tests	Findings
Baker et al., 2015	64 HIV+ individuals	CS	Washington, USA (WUSTL Infectious Disease Clinic and AIDS Clinical Trials Group)	N/R	Age: 37.96 ± 12.92, Education: 13.17 ± 2.37, Gender: 66% Male, Race/ethnicity: 76% African American, 24% White	Mean duration: 8.9 years	Recent CD4: 539.50; Nadir CD4: 227.00; Recent plasma VL log10: 1.30	All on CART for > 3 months	CPE 2010	Continuous and categorical (CPE ≥ 7 vs. <7)	**Domains:** Memory, psychomotor speed, executive function, and verbal function **NP Tests**: Hopkins Verbal Learning Test-Revised (HVLT-R), Trailmaking Test-Part A and B (TMT-A, TMT-B), and Controlled Oral Word Association Test (COWAT)-Animal Fluency.	CPE 2010 not associated with global, processing speed, verbal learning, executive functioning, verbal fluency
Carvalhal et al., 2016	417 HIV+ participants	CS	Ontario, Canada (Ontario HIV Treatment Cohort Study)	1996-?	Age: 46.8 ± 9.7; Education 13.9 ± 2.6; Gender: 81% male; Race: 62% white	Years since diagnosis: 11.2 ± 6.4	Current CD4 >500: 52%; Nadir CD4 <200: 62%; Plasma viral load <50: 92%	Years since first ARV treatment: 7.6 ± 5.3; Participants on 3 cART drugs for at least 90 days prior to assessment.	CPE 2010	continuous and categorical (CPE 5, 6, 7, or ≥ 9)	**Domains**: Learning/memory, processing speed, working memory, motor functioning NP Tests: HVLT, BVMT, WAIS Digit Symbol Test, WAIS Spatial Span Test, Grooved Pegboard Test	Higher CPE 2010 associated with better global NP
Casado et al., 2014	229 HIV+ individuals	CS	Madrid, Spain	2011 - 2012	Age: 44.6 (IQR 38-51); Gender: 77% male; Race/ethnicity: 86% white, 1% African American, 13% Hispanic; Education: 28% college; 29% high school, 43% less than high school	Median Duration: 12.18 months	Recent CD4: 325 (210-509), Nadir CD4: 213 (100-311), plasma VL log10: 4.2 (1.7-5.1); plasma VL <50: 32%	Time on current ARV: 33 months (14-49); Time on any ARV: 138.1 (39.1-187)	CPE 2010	Categorical (CPE ≥ 7 vs. <7)	**Domains:** only Global **NP Tests**: TMT-A, TMT-B, Digit Symbol Test, and Grooved Pegboard	Higher CPE 2010 associated with better global NP (trend only)
Ciccarelli et al., 2013	101 HIV+ individuals, 30 HIV- controls	CS	Rome and Siena, Italy	2008 - 2010	Age: 47 (IQR 42-52), Gender: 65% male Education: 13 (IQR 8-13)	Median duration: 11.8 years (7.2-16.9)	Current CD4: 620 (454-827), Nadir CD4: 171 (62–264)	Time on current ARV > 12 months: 74%; Time on any ARV: 9.6 years (5.1-11.8)	CPE 2008, CPE 2010	Categorical: CPE 2008 ≥1.5 vs. < 1.5; CPE 2010 ≥ 6 vs. < 6	**Domains:** Memory, attention, executive functioning, processing speed, language **NP Tests**: Rey's words, Rey-Osterrieth Complex Figure Test (RCFT), WAIS Digit Span, WAIS Spatial Span, Stroop Test, TMT-B, Drawings and Double Barrage, WAIS Digit Symbol, COWAT Letter Fluency	Higher CPE 2010 associated with better global (HAND), attention, memory<br><br>CPE 2008 not associated with NP
Cross et al., 2013	111 HIV+ individuals	Long.: baseline, 1 year follow-up	Cape Town, South Africa	N/R	N/R	N/R	Recent CD4 count: 162.32, plasma VL <50: 100%	Duration of ARV at least 10 months: 100%	CPE 2010	Categorical: CPE ≤7, CPE > 7	**Domains:** only Global **NP Tests**: Finger Tapping Test, Grooved Pegboard, HVLT-R, Brief Visuospatial Memory Test-Revised (BVMT-R), Mental Alternation Test, WMS-III Digit Symbol Coding, TTMT-A, Color Trails, Stroop Test, Wisconsin Card Sorting Test (WCST), RCT, and COWAT-Animal and Fruit/Vegetable	CPE 2010 not associated with global NP
Cysique et al., 2009	37 HIV+ individuals	Long.: 12, 24, 36, and 48 weeks after CART initiation	San Diego, California, USA (HNRC)	1996 - 2006	Age: 39.7 ± 7.3; Education: 13.6 ± 3.2; Gender: 86.5% Male; Race/ethnicity: 73% White, 24% Hispanic, and 3% African-American	N/R	Nadir CD4: 106.9 ± 102.4, CD4 at baseline: 195.6 ± 161.9, Plasma HIV RNA (log10) Mean (IQR): 4.9, (4.01-5.6), CSF HIV RNA	Participants were either untreated (38%) or failing therapy (i.e., plasma HIV RNA > 5,000; 62%) and prescribed new CART regimen at baseline	CPE 2008	Categorical (CPE ≥ 2 vs. CPE < 2)	**Domains:** Global only **NP Tests**: Grooved Pegboard, Paced Auditory Serial Addition Test (PASAT), Trailmaking Test Parts A & B, COWAT Letter Fluency	Higher CPE 2008 associated with better global NP

							(log10) Mean (IQR): 3.6 (2.9-4.3)					
Ellis et al., 2014	49 HIV+ individuals	CS	California (San Diego, San Francisco), Maryland, Missouri, New York, USA	2007 - 2012	Age: 44.9 ± 10.08, Education: 12.24 ± 2.17, Gender: 80% Male; Race/ethnicity: 39% White, 51% Black, 8% Hispanic 2% Other	N/R	Current CD4 = 242, Nadir CD4 =175<br><br>**CNS Targeted (n= 26)**: Plasma VL = 4.2 (IQR 1.7-5.9), CSF HIV RNA = 3.1 (IQR 1.7-4.6)<br>**non-CNS Targeted (n= 23)**: Plasma VL = 3.5 (1.7-6.2), CSF HIV RNA = 3.1 (1.7-5)	Prior ARV treatment = 69% (Participants were either untreated or changing regimen)	CPE 2008	Categorical	**Domains:** Executive Functioning, Processing Speed, Verbal Fluency, Learning/Memory, Attention/Working Memory, Motor.<br>**NP Tests**: Wisconsin Card Sorting Test-64-item (WCST-64), TMT-A, TMT-B, DKEFS Color-Word Test, WAIS-III Digit Symbol and Symbol Search, COWAT-Animals, Action Fluency, HVLT-R, BVMT-R, WMS-III-Spatial Span, PASAT, Grooved Pegboard	Higher CPE 2008 associated with better global (trend only)
Fabbiani et al., 2015	215 HIV+ individuals	CS	Rome and Galatina, Italy	2009 - 2013	Age: 44 (38-50), Education: 13 (8-13), Gender: 77% Male; Race/ethnicity: 9% non-Italian born	Median duration: 9.9 years (IQR 3.5 -16.3)	Current CD4 = 545 (IQR 398–697), Nadir CD4 = 211 (IQR 87–296), plasma VL <50 = 88%	Time on current ARV regimen: 16 months (IQR 7–34)	CPE 2010	Continuous; composite score accounting for viral resistance also computed	**Domains:** global, memory, attention, processing speed, motor functioning, language<br>**NP Tests**: Rey's words, WAIS Digit Span, WAIS Digit Symbol, Grooved Pegboard, Letter Fluency	Higher CPE 2010 associated with better global, only after accounting for resistance<br><br>Higher CPE 2010 associated with better memory, motor (trend only)<br><br>CPE 2010 not associated with attention, processing speed, language
Force et al., 2021	31 HIV+ individuals	Long.: baseline, 48 and 96 weeks	France	N/R	Age: 54 (47–58), Education: 11 (9–14), Gender: 84% Male; Race/ethnicity: N/R	Median duration: 19 years (8–24)	Current CD4 = 619 (IQR 396–761); Nadir CD4 = 165 (55–281), plasma VL < 5 = 52%, CSF VL < 5 = 71%	Currently on CART = 100%; Duration of current regimen = 3 years (IQR 2–5); Duration on any ARV regimen = 14 years (IQR 6–17)	CPE 2010	Categorical; score accounted for viral resistance	**Domains:** global only<br>**NP Tests:** Grooved Pegboard; GREFEX Verbal Fluency; CVLT; WAIS-III Digit Span; modified PASAT-60 items; WAIS-III Digit Symbol; TMT A&B; WCST	Higher CPE associated with better NP (HAND; GDS)
Heaton et al., 2010	1,555 HIV+ individuals	CS	Maryland, New York, California, Texas, Washington, Missouri (CHARTER studies)	2003 - 2007	Age = 43.2 ± 8.5, Education = 12.5 ± 2.5, Gender: 23% Female, Race/ethnicity = 39% White, 49% African American, 9% Hispanic 3% Other	N/R	Current CD4 = 420 (IQR = 262-603), Nadir CD4 = 174 (IQR = 49-300), Detectable plasma VL = 59%, Detectable CSF VL = 34%	Currently on CART = 71% Duration of current regimen = 11 months (IQR = 4-27)	CPE 2008	Continuous	**Domains:** global only<br>**NP Tests**: Digit Symbol-Coding, TMT-A & B, WMS Logical Memory, RAVLT, BVMT-R, Boston Naming Test, COWAT-Letter and Semantic Fluency, WAIS Bock Design	CPE 2008 not associated with global NP
Heaton et al., 2015	436 HIV+ individuals	Long.: visits every 6 months, 4–7 study visits (16–72 months of follow-up,	Maryland, New York, California, Texas, Washington, Missouri (CHARTER studies)	2003 - 2007	Age = 43.9 ± 8.4, Education = 12.9 ± 2.5, Gender = 80% Male, Race/Race/ethnicity = 43% White, 44% Black, 11% Hispanic, 2% Other	N/R	Current CD4 count = 459 (IQR 289-644), Nadir CD4 = 184 (IQR 49-320), Undetectable plasma VL plasma = 41% (58% if on ART),	Currently on CART: 70%; Duration of current regimen =18.0 months ± 21.2	CPE 2008	Continuous	**Domains:** global only<br>**NP Tests**: Digit Symbol-Coding, TMT-A & B, WMS Logical Memory, RAVLT, BVMT-R, Boston Naming Test, COWAT-Letter and Semantic Fluency, WAIS Bock Design	CPE 2008 not associated with global NP

		mean: 35 months)					Undetectable HIV in CSF 66% (85% if on ART)					
Kahouadji et al., 2013	54 HIV+ individuals	CS	Paris, France	2003 - 2006	Age = 41.7 ± 11.1, Gender = 35% Female, Education = 10.1 ± 3.7; Race/ethnicity not reported (all participants resided in France)	Mean duration: 7.3 years ± 11.4	Current CD4 count = 460.4 ± 192.9	Patients continued to receive their ARV treatment for 3 years or switched once to a new treatment with the same CPE score	CPE 2008	Continuous; Categorical by tertile (CPE ≤1; 1 - 2.5; ≥2.5)	**Domains:** executive function, memory **NP Tests**: Frontal Assessment Battery (FAB), Clock Drawing Test, 5 Words Test, and Praxis Ability Scale	Higher CPE 2008 associated with worse executive function<br><br>CPE 2008 not associated with memory
Keutmann et al., 2017	128 HIV+ individuals and 152 HIV- individuals	CS	Chicago, Illinois, USA	N/R	Age: 47.43 years ± 7.84; Education: 12.19 ± 2.02; Gender: 59% male, 41% female; Race/ethnicity: 88% African American	N/R	Reported by subgroup, not reported for entire sample	N/R	CPE 2008	continuous	**Domain**: Learning/Memory **NP Tests**: BVMT-R	Higher 2010 CPE scores associated with better memory
Lawler et al., 2011	140 (60 HIV+ individuals, 80 HIV- demo-matched controls)	CS	Gaborone, Botswana	2009 - 2009	Age = 37.5 ± 6.2, Education = 8.6 ± 4.2, Gender = 48.3% Male; Race/ethnicity not report (all participants resided in Botswana)	Mean duration: 4.6 years ± 2.5	Current CD4 count = 416.4 ± 198.5, Nadir CD4 = 97.6 ± 49.9, Viral Load < 400 = 93.3%	CART treatment duration = 4.1 years ± 2.1, On >3 ARV medications = 100%;	CPE 2008	Continuous	**Domains:** Processing speed, verbal learning/memory, executive function, language/verbal fluency, and motor function **NP Tests:** WAIS-III Digit Symbol Coding; Verbal Fluency for Action; Botswana Auditory Verbal Learning Test (BAVLT); TMT-A; Color Trails 2; Grooved Pegboard	CPE 2008 not associated with processing speed, memory, executive function, motor, verbal fuency
Marra et al., 2009	79 HIV+ individuals	Long.: baseline, 24, and 52 weeks after initiating CART	Multiple sites through ACTG (no further details)	N/R	Age = 39 (IQR 33-45), Education = 13 (IQR 12-15), Gender = 17% Female; Race/ethnicity = 51% Non-white	N/R	Current CD4 = 111 (IQR 49-224), Plasma HIV VL og10 = 4.86 (IQR 4.55-5.30), CSF VL log10 = 3.33 (IQR 2.50-3.85)	All participants initiating a new cART regimen or changing existing regimen; all on ≥ 3 ARVs	CPE 2008	Categorical (CPE ≥ 2 or < 2)	**Domains:** Global only **"Short battery"NP Tests**: Timed Gait, Grooved Pegboard, WAIS Digit Symbol, Finger Tapping **"Long battery" NP Tests**: Timed Gait, Grooved Pegboard, WAIS Digit Symbol, Finger Tapping, RAVLT, TMT, Finger Tapping, Basic Choice Reaction Time (CalCAP), and Sequential Reaction Time.	Higher CPE 2008 associated with worse global NP
Sanford et al., 2018	79 (48 HIV+ individuals, 31 HIV- controls)	Long.: baseline, approximately 2 years follow-up	St. Louis, Missouri, USA	2011 - 2016	Age = 47.7 ± 13.2, Educational = 13.3 ± 3.4, Gender = 52% Male, Race/Race/ethnicity = 31% White, 69% African-American	Median duration (IQR) = 13.5 years (5.2-20)	Nadir CD4 count = 190 (IQR 57-300), Current CD4 count = 630 (IQR 486, 881), plasma HIV VL <50 = 100% of sample	N/R	CPE 2008	Continuous	**NP Tests**: TMT, Digit Symbol Substitution Task (DSST), WAIS Letter-Number Sequencing, COWAT Letter Fluency, Action Fluency, HVLT-R	CPE 2008 not associated with attention, processing speed, memory, executive function
Shikuma et al., 2012	139 HIV positive individuals	CS (CPE analyses) and Long.: 1 year, 2 year, and 3 year follow-up	Hawaii, USA (Hawaii Aging with HIV Cohort)	2001 - 2006	Age: Younger group = 37.3 (IQR 33.4-39.3), Older group = 53.7 (IQR 51.1-57.9); Education = 12 (12-16), Gender = 85%, Male, 15% Female, Race/ethnicity = 61% White, 28% Asian/Pacific Islanders, 11% Other	Median duration (IQR): 9.6 years (5.1-15)	Nadir CD4: 150 (50-300) Current CD4: 510.0 (318.0-647.0) Log HIV RNA <50 copies/ml: 66.19, Log HIV RNA in those with >50 copies/ml: 3.6 (2.6-4.7)	Duration of current ARV regimen at entry, median (IQR): 2.1 years (1.0-3.6).	CPE 2008 and CPE 2010	continuous	Domains: global only **NP Tests**: Timed Gait, Grooved Pegboard, Trail Making Test-Parts A and B, WAIS-R Digit Symbol, Choice and Sequential Reaction Time from CalCap.	CPE 2010 not associated with global NP
Simoni et al., 2010	100 HIV+ individuals	CS	Lausanne and Geneva, Switzerland	N/R	Age = 47.5 ± 9.55, Education = 73% completed high school, Gender = 72% Male,	Duration of HIV infection = 12.1	Nadir CD4 = 170.6 ± 132.7, Current CD4 = 597.3 ± 262.1	Duration of Current ARV regimen: 2.5 years ± 2.4	CPE 2008	Continuous	**Domains:** Global only **NP Tests**: Cambridge Neuropsychological Test Automated Battery (CANTAB) Reaction time, Rapid Visual Information	CPE 2008 not associated with global NP

					Race/ethnicity = 70% White	years ± 6.5					Processing, and Spatial Working Memory subtests, TMT	Higher CPE 2008 associated with less severe HAND diagnosis
Smurzynski et al., 2011	2,636 HIV+ individuals	Long.: NC evaluated every 48 weeks	26 US States (ACTG ALLRT cohort)	1997 - 2008	Age = 40 (IQR 34, 47), Education = 14 (IQR 12, 16) Gender = 83% Male 17% Female, Race/ethnicity = 53% White, 28% Black, 20% Hispanic	N/R	Current CD4 = 243.5, Nadir CD4 = 182 Current plasma VL = 43,036	69% ARV naive at baseline	CPE 2008	Continuous	**Domain:** Global only<br>**NP Tests**: Trail Making Test (Trail A & B) and the Wechsler Adult Intelligence Scale-Revised (WAIS-R) Digit Symbol Test.	Higher CPE 2008 associated with better global NP (only with 3+ ARVs)
Tozzi et al., 2009	85 HIV+ individuals	Long.: every 3 months (30 time points)	Rome, Italy	1999 - 2006	Age = 39.3 ± 8.3, Education = 11.4 ± 3.6, Gender = 74% Male	Duration: 65.8 months ± 59.0	Nadir CD4 = 181.6 ± 174.8, Current CD4 = 292.9 ± 291.3, Plasma LV log10 = 4.14 ± 1.29	95% ARV naive at baseline; prescribed 3-drug CART regimen	CPE 2008	Continuous	**Domains:** global, attention/concentration, processing speed, mental flexibility (EF), memory, motor function<br>15 test standardized NP battery, individual tests not reported	Higher CPE 2008 associated with better global NP, attention, processing speed, executive functioning, motor<br><br>CPE 2008 not associated with memory
Vassallo et al., 2014	96 HIV+ individuals	Long.: Baseline, approximately 2-year follow-up	Nice, Italy	2007 - 2011	Age = 48 ± 11, Gender = 80% Male	Duration: 12 years ± 7.5	Nadir CD4 = 265 ± 182, Current CD4 = 551 ± 272, plasma VL = 10,760 ± 34,828, plasma VL <40 = 58%	Duration on current ARV regimen = 24.6 months ± 20.5	CPE 2010	Continuous	**Domains:** Global only<br>**NP Tests**: Grober and Buschke test, 'Four seconds' test, Paced Auditory Serial Addition Task (PASAT), Stroop test, Modified Card Sorting test, Finger tapping, Timed alternating hand sequence test), Verbal Fluency, and Protocole Montral-Toulouse d'Evaluation des Gnosies Visuelles	Higher CPE 2010 associated with better global NP (HAND)
Wilson et al., 2013	118 HIV+ individuals, 310 HIV− individuals	CS	Chicago, Illinois, USA	N/R	Age: 43.45 years ± 6.57; Education: 11.63 ± 2.07; Gender: 64% Male, 36% Female; Race/ethnicity: 88% African American		Undetectable viral load: 64% of sample	N/R	CPE 2010	continuous and categorical (CPE ≤ 7 vs. CPE > 7)	**Domain**: Learning (Procedural)<br>**NP Tests**: Rotary Pursuit Task; Weather Prediction Task	CPE 2010 not associated with learning/memory
Wright et al., 2010	292 HIV+ individuals	CS	47 sites in Australia, North America, Brazil, and Thailand (SMART study)	2005 - 2006	Age = 40 (IQR 35-45.5), Education = 54% less than 12 years, Gender = 42% Female, Race/ethnicity = 51% Asian, 20% Black, 30% Other	N/R	Nadir CD4 = 225 (IQR 158-296), Current CD4 count = 536 (IQR 437-693), plasma VL < 400 = 88%	Duration on any ARV regimen = 4 years (IQR 3–6)	CPE 2008	Continuous	**Domain:** "Global" (based only on motor and processing speed)<br>**NP Tests**: Grooved Pegboard, Finger Tapping, Color Trails, Timed Gait	CPE 2010 not associated with "global" NP (motor, processing speed)

Note: N/R = not reported; CS = Cross-sectional; Long. = Long.
